# Preoperative adjuvant therapy for locally advanced and recurrent/metastatic gastrointestinal stromal tumors: a retrospective study

**DOI:** 10.1186/s12957-020-01840-9

**Published:** 2020-04-07

**Authors:** Jing Qi, He-Li Liu, Feng Ren, Sheng Liu, Wei Shi, Wei-Hang Liu, Gao-Qiang Cai, Guo-Qing Liao

**Affiliations:** 1 Department of Gastrointestinal Surgery, Xiangya Hospital of Central South University, Changsha, 410008 Hunan People’s Republic of China; 2 Department of Geriatric Surgery, The Second Xiangya Hospital, Central South University, Changsha, 410011 Hunan People’s Republic of China; 3 Department of Radiology, Xiangya Hospital Central South University, Changsha, 410008 Hunan People’s Republic of China

**Keywords:** Gastrointestinal stromal tumor, Preoperative treatment, Imatinib mesylate, Retrospective study

## Abstract

**Background:**

Preoperative imatinib mesylate therapy for gastrointestinal stromal tumors (GISTs) is controversial. This study aimed to explore the clinical efficacy and optimal duration of preoperative imatinib mesylate (IM) therapy in patients with locally advanced and recurrent/metastatic GISTs.

**Methods:**

We retrospectively examined patients who received preoperative imatinib mesylate therapy from January 2013 to December 2018 at Xiangya Hospital, Central South University and the Second Xiangya Hospital of Central South University, China. Clinical data, including the results of tests for mutations in *KIT* and *PDGFR*, findings from regularly conducted re-examinations, abdominal-enhanced computed tomography/magnetic resonance imaging data, responses to imatinib, progression-free survival, and overall cancer-specific survival, were recorded.

**Results:**

A total of 25 patients were enrolled in our study, including 18 with a locally advanced GIST and 7 with recurrent or metastatic GISTs. Their ages ranged from 22 to 70 years (M:F = 1.6:0.9), with a mean age of 50.48 ± 12.51 years. The tumor locations included the stomach (56.0%), rectum (16.0%), enterocoelic/retroperitoneal sites (12.0%), and the small intestine (12.0%). Based on testing for mutations in *KIT* and *PDGFR*, 22 patients received 400 mg/day *KIT*, and 3 patients received 600 mg/day *PDGFR*. The median duration of preoperative IM therapy was 8.96 ± 4.81 months, ranging from 3 to 26 months. According to the Choi criteria, 24 patients achieved a partial response (PR), and 1 patient had stable disease (SD). All patients underwent surgery after preoperative IM therapy, and no postoperative complications appeared. The 2-year PFS and 5-year PFS were 92% and 60%, respectively, and the total 5-year cancer-specific survival (CSS) was 92%.

**Conclusion:**

Preoperative imatinib therapy is feasible for locally advanced and recurrent/metastatic GISTs and can effectively shrink the tumor size, allow organ sparing, and avoid extensive organ resection. Moreover, the optimal duration of preoperative IM therapy in patients with locally advanced and recurrent/metastatic GISTs was 8.96 ± 4.81 months, ranging from 3 to 26 months, and gastric GISTs had a better response to preoperative IM therapy than did non-gastric GISTs.

## Backgrounds

Gastrointestinal stromal tumors (GISTs) are the most common primary mesenchymal tumors occurring throughout the gastrointestinal tract, with an annual incidence of approximately 7 to 15 per million inhabitants per year and accounting for approximately 1 to 3% of all malignant gastrointestinal tumors [1, 2]. GISTs occur most frequently in the stomach and small intestine [3]. Total tumor resection is the only curative option and the basis of GIST treatment. However, up to 20% of GIST patients have metastases at diagnosis [4], most commonly in the abdominal cavity, liver, lungs, bones, and other rare organs. Lymph node metastases are uncommon clinical features in GISTs, except for pediatric GISTs, pediatric-type GISTs in young adults, and syndromic GISTs [5].

The diagnosis of GISTs depends on the immunohistochemistry of tumor tissues and gene mutation analysis. Most GISTs stain for *KIT* protein (CD117; 95%) [6] and anoctamin-1 (DOG-1; 98%) [7], but most do not stain for desmin, a biomarker of smooth muscle tumors. Moreover, approximately 75% of GISTs have a mutation in *KIT* occurring in exons 9 (8%), 11 (90%), 13 (1%), and 17 (1%), and 10% to 20% of GISTs have a mutation in platelet-derived growth factor receptor A (*PDGFRA*) occurring in exons 12, 14, and 18 [8, 9], which encode type III receptor tyrosine kinase and are the cornerstone for the receptor tyrosine kinase inhibitors (RTKIs) applied in GIST patients [10].

Before receptor tyrosine kinase inhibitors (RTKIs) were applied in GISTs, the survival rate was still poor despite the resection of primary GIST tumors [11]. Imatinib mesylate (IM), a typical type of RTKI, dramatically improves the prognosis of GISTs, especially in metastatic/recurrent GISTs [12]. Surgery combined with adjuvant IM has greatly improved the prognosis of patients with intermediate or high-risk categories according to the revised National Institutes of Health (NIH) guidelines (2008). Even so, when the tumor sizes are too large or the tumor locations are special (e.g., cardia of the stomach, duodenal nipple, or rectum), up-front surgical resection should be deeply considered because it might excise surrounding organs and lead to an extensive R0 organ resection procedure (e.g., total gastrectomy, pancreaticoduodenectomy, or abdominoperineal excision of the rectum); such treatment would eventually lead to permanent changes in the patient’s lifestyle or increase the operative morbidity. In that case, imatinib mesylate (IM) is the only TKI that has been studied as adjuvant therapy at a dosage of 400 mg/day, which aims to shrink the tumor size and preserve the surrounding organs to the greatest extent [13, 14].

However, the optimal duration of preoperative imatinib is still unknown, and it is always recommended for 6 to 12 months to shrink the tumor size after IM initiation. Multidisciplinary team members should cooperate to monitor the response to IM and determine the optimal operation time for those patients. In addition, both 6 months of preoperative IM therapy and surveillance when tumor size shrinkage is detected by consecutive radiographic imaging in metastatic/recurrent GISTs are suggested [15, 16], but the survival benefit from an operation following IM therapy cannot be determined. Hence, our study aimed to explore the clinical efficacy and the optimal duration of preoperative IM therapy in patients with locally advanced and recurrent/metastatic GISTs.

## Methods

### Patient selection

GIST patients were collected through a pathology information system and were diagnosed from January 2013 to December 2018 at Xiangya Hospital, Central South University and the Second Xiangya Hospital of Central South University. Further clinical information and clinical follow-up data were collected by our clinical system and communication with patients. The eligibility criteria in this study included the following: (1) patients were pathologically confirmed as having locally advanced or recurrent/metastatic GISTs by morphology and immunohistochemical staining, and the specimens were obtained by fine-needle biopsy, surgery, or endoscopy; (2) GISTs were assessed as unresectable or as needing extensive organ resections for R0 resection, as evaluated by our multidisciplinary team including experienced oncologists, surgeons, pathologists, and radiologists; (3) all GIST patients had no history of radiotherapy and chemotherapy before taking preoperative IM therapy; and (4) the Eastern Cooperative Oncology Group performance status score was 0 or 1. The exclusion criteria were as follows: (1) GISTs showed primary resistance to IM by mutation analysis or radiographic imaging, (2) patients could not tolerate operation or IM therapy, and (3) patients refused to take preoperative IM therapy and strongly required surgery directly. This retrospective study was supported by the Medical Ethics Committee of Xiangya Hospital, Central South University (No. 201909812), and due to the retrospective nature of the study, informed consent was waived.

### Preoperative procedure

The preoperative procedure was managed by a multidisciplinary team comprising oncologists, surgeons, pathologists, and radiologists. GIST specimens were collected by fine-needle biopsy, surgery, or endoscopy, formalin-fixed, paraffin-embedded, and diagnosed by microscopic morphology and immunohistochemistry for CD117, DOG-1, and desmin. The initial dosage of IM was based on the results of the mutation analysis. Patients who had *KIT* exon 9 mutations were administered a dosage of 600 mg once daily, others received 400 mg once daily, and close monitoring was performed to assess them by contrast-enhanced computerized tomography (CT) scans or contrast-enhanced magnetic resonance imaging (MRI) scans. The multidisciplinary team discussed and evaluated the optimal timing for surgical resection. IM therapy was discontinued immediately before surgical resection.

### Therapeutic assessment

Contrast-enhanced CT scans or contrast-enhanced MRI scans were performed to monitor tumor progression; initial scans were conducted before IM therapy, and follow-up CT or MRI scans were performed every 3 months. The response of the target lesions was evaluated according to the Choi criteria [17]. The plateau response was defined at the point in the sum of diameters of target lesion shrinkage between 10% decrease and 10% increase, with a decrease in tumor density (HU) of less than 15%, detected by two consecutive radiographic images. The total response was classified according to Choi criteria as complete response (CR), partial response (PR), progressive disease (PD), and stable disease (SD).

### Operation timing and postoperative therapy

The optimal operation timing was as follows: (1) two consecutive radiographic images indicated that the tumor response was at the plateau; (2) the multidisciplinary team discussed and evaluated a meaningful downstaging, including converting the total gastrectomy to local tumor resection, converting the pancreaticoduodenectomy (Whipple operation) to local excision and converting abdominoperineal resection with permanent end colostomy to a sphincter-sparing transanal resection; (3) patients could not tolerate the side effects and directly demanded surgical treatment; and (4) patients exhibited massive alimentary tract bleeding or gastrointestinal perforation that required an emergency operation.

Postoperative IM adjuvant treatment at a dosage of 400 mg/day or 600 mg/day was restarted as soon as the patients were able to tolerate oral medications, patients with non-metastatic tumors that were locally advanced or in special locations were treated with R0 resection and continued imatinib adjuvant treatment for 36 months in total; patients with recurrent/metastatic GISTs continued imatinib adjuvant treatment until the follow-up period ended. The last follow-up was considered performed in October 2019 or prior to patient death for any cause.

### Statistical analysis

The counting data are shown as percentages, and measurement data are shown as the mean or median with standard deviation (SD). The chi-squared test was used to evaluate the relationship between optimal duration and the clinicopathological characteristics, and Student’s *t* test or one-way ANOVA was used for comparison between different groups. Differences were considered statistically significant at *P* < 0.05. Progression-free survival (PFS) and cancer-specific survival (CSS) were obtained by the Kaplan-Meier method. Statistical analysis was performed using SPSS V19.0 (SPSS, Inc., USA).

## Results

### Demographic data and clinicopathologic data

**Fig. 1:**
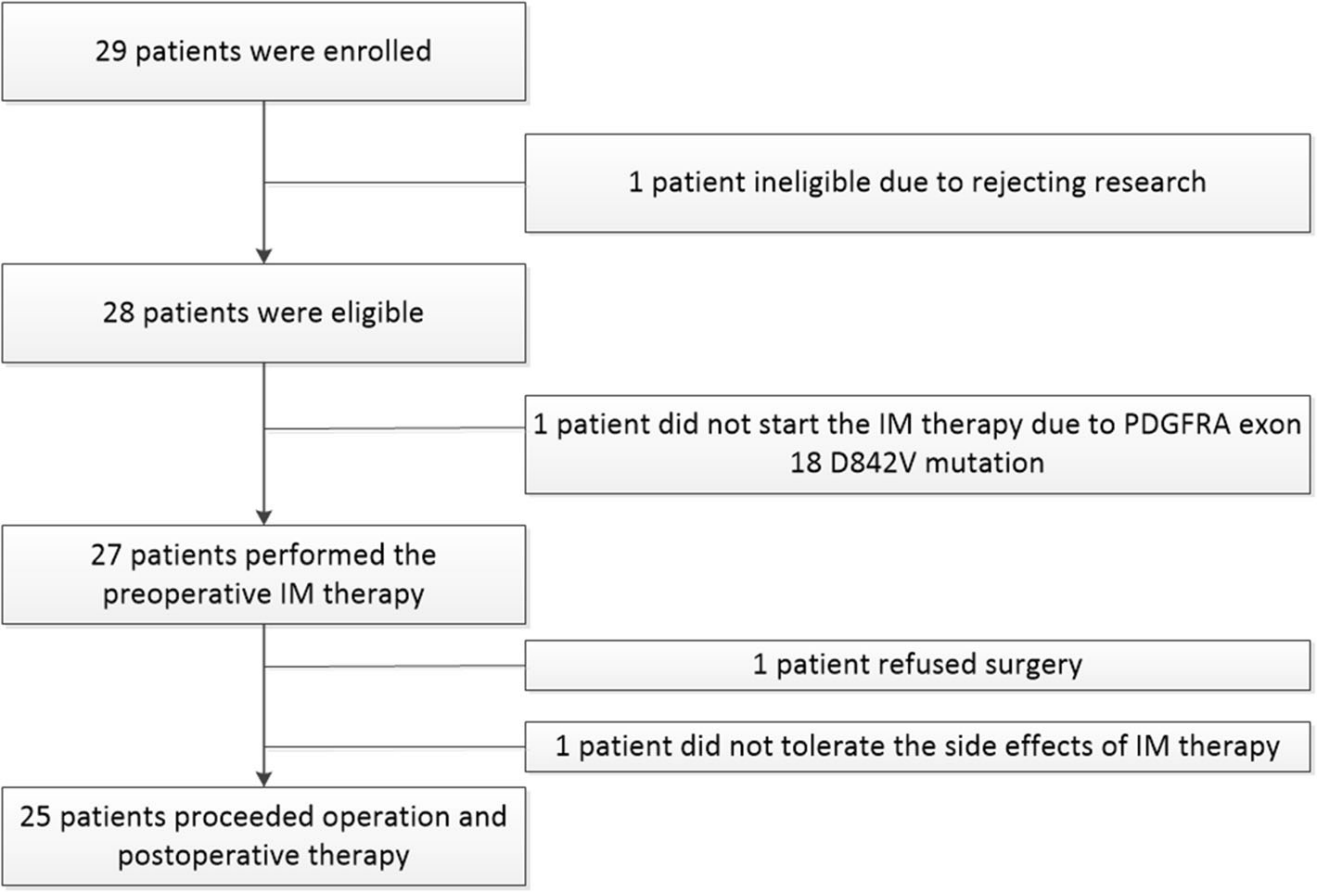
Study profile. PDGFRA, platelet-derived growth factor receptor alpha; IM, imatinib mesylate

A total of 29 patients were enrolled in our study; however, one patient refused to join our research, and one patient did not undergo operative IM therapy because the gene mutation analysis showed a *PDGFRA* exon 18 D842V mutation. One patient did not tolerate the IM side effects and refused subsequent therapy, and one patient refused to undergo surgery until the end of the follow-up. Therefore, an effective analysis of preoperative IM therapy was conducted in the remaining 25 patients (Fig. 1). The analytic cohort of 25 patients had a mean age of 50.48 ± 12.51 years, ranging from 22 to 70 years, comprising 16 (64.0%) men and 9 (36.0%) women. Of all patients, 18 had locally advanced GISTs, and 7 had recurrent or metastatic GISTs. The pretreatment maximum diameter of each GIST was measured by CT and MRI scans, with an average of 11.48 ± 4.46 cm, ranging from 4.5 to 22.4 cm. The analytic cohort tumors were located in the stomach (56.0%), small intestine (16.0%), rectum (16.0%), and enterocoelic/retroperitoneum (12.0%). All patients underwent gene mutation analysis, and the results showed that 3 patients had *KIT* exon 9 mutations, 18 patients had *KIT* exon 11 mutations, 1 patient had *KIT* exon 11 and exon 13 mutations, one patient had *KIT* exon 17 mutations, and 2 patients had no mutations in either *KIT* or *PDGFRA*, which were called wild-type GISTs (WT-GISTs). More clinical and pathological data are shown in Table 1 and Table S1.

**Table 1:** Characterization of the study group prior IM therapy

Characteristics		Patients *n* (%) or mean ± SD
Median age, years [range]		50.48 ± 12.51 [22–70]
Gender	Male	16 (64.0%)
	Female	9 (36.0%)
Classify of preoperative therapy	IM therapy in locally advanced GISTs	18 (72.0%)
	IM therapy in recurrent/metastasis GISTs	7 (28.0%)
Primary localization	Stomach	14 (56.0%)
	Duodenum	4 (16.0%)
	Jejuno-ileum	1 (4.0%)
	Rectum	4 (16.0%)
	Pelvic/retroperitoneal	2 (8.0%)
Dose of IM therapy	400 mg/day	22 (88.0%)
	600 mg/day	3 (12.0%)
Duration of preoperative therapy, months [range]		8.96 ± 4.81[3–26]
Preoperative maximal dimension, cm [range]		11.48 ± 4.46 [4.5–24.4]
Gene mutation, wild type vs. mutation	*KIT* in exon 9	22 vs. 3
	*KIT* in exon 11	6 vs. 19
	*KIT* in exon 13	24 vs. 1
	*KIT* in exon 17	24 vs. 1
	*PDGFRA* in exon 12	25 vs. 0
	*PDGFRA* in exon 18(D842V)	25 vs. 0
Numbers of lesions	Single	19 (76.0%)
	Multiple	6 (24.0%)
Response to IM therapy (according to Choi criteria)	PR	24 (96.0%)
	SD	1 (4.0%)
	PD	0 (0.0%)
Margin status	R0	24 (96.0%)
	R1	0 (0.0%)
	R2	1 (4.0%)
ECOG performance status	0	18 (72.0%)
	1	7 (28.0%)

### Clinical efficacy to IM therapy

**Fig. 2:**
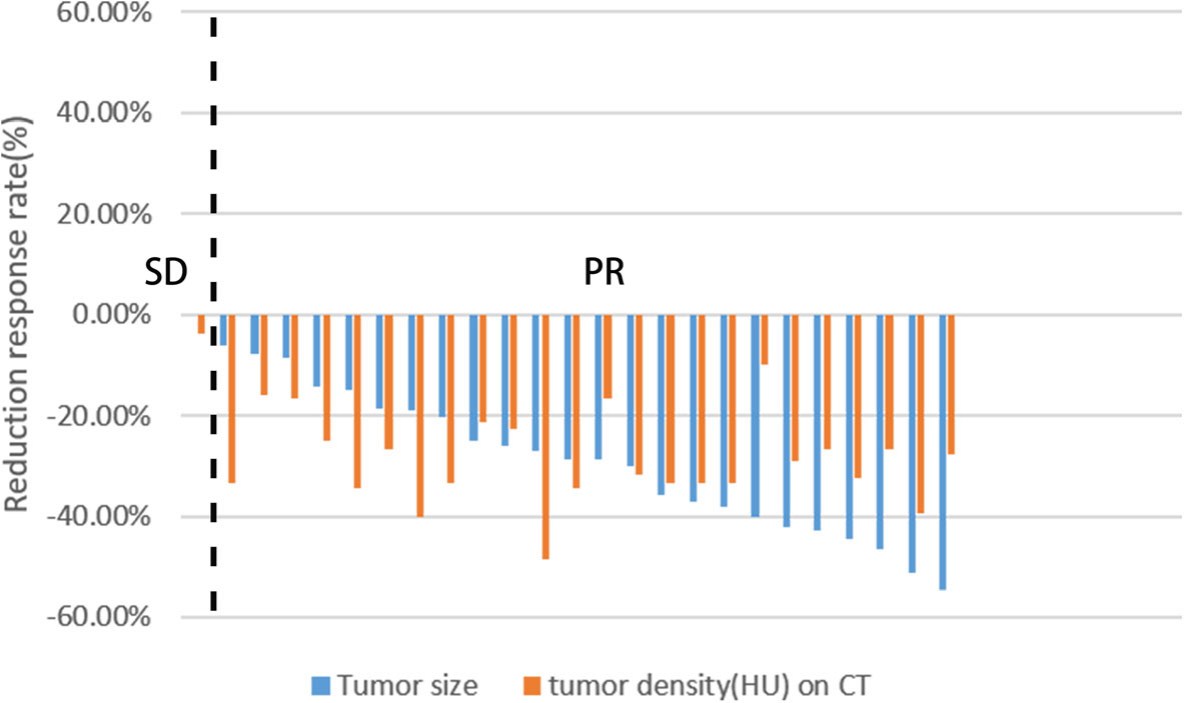
Waterfall plot of the ranked best tumor shrinkage after preoperative IM therapy

Based on the mutation analysis, 3 patients with *KIT* exon 9 mutations were administered the dosage of 600 mg once daily, and the other 22 patients were administered at the dosage of 400 mg once daily. According to the Choi criteria, 24 patients achieved a partial response (PR), and 1 patient developed a stable disease (SD) and received surgery immediately after completing 4 months of preoperative IM treatment; the mutation analysis showed a wild-type GIST (Fig. 2). All patients underwent surgery after preoperative IM therapy, and 24 patients achieved effective shrinkage of the tumor size to various extents, with sizes decreasing from 11.48 ± 4.46 cm to 8.24 ± 4.02 cm (*P* ≤ 0.001). All patients underwent surgery after preoperative IM therapy. Fourteen gastric GIST patients underwent preoperative therapy. One of them achieved an SD response and underwent proximal gastrectomy (R0 resection) after 4 months of preoperative IM treatment. Ten patients who were assessed to perform total gastrectomy before IM therapy by our multidisciplinary team finally underwent partial gastrectomy (R0 resection) after preoperative IM treatment, including 3 patients who underwent laparoscopic partial gastrectomy and 3 patients who were assessed for unresectable tumors because of a large recurrent tumor size or metastasis to the liver and finally underwent partial gastrectomy (R0 or R2 resection). Two patients in five small intestine GIST patients finally underwent pancreaticoduodenectomy (R0 resection) because the tumors were too near to the duodenal papilla, 1 patient who was assessed to perform pancreaticoduodenectomy before IM therapy finally underwent partial duodenum resection (R0 resection) after preoperative IM treatment, and 2 patients with small intestine metastatic/recurrent GISTs who were considered unresectable before IM therapy underwent local resection of the liver and tumor resection combined with partial small bowel resection (R0 resection). Four rectal GIST patients underwent preoperative therapy, two finally underwent laparoscopic abdominoperineal resection (R0 resection), and the other 2 patients who were assessed for abdominoperineal resection finally underwent transanal minimally invasive surgery and sphincter-sparing resection (R0 resection), respectively. The remaining two pelvic/retroperitoneal GIST patients who received preoperative therapy and had been considered unresectable before IM therapy finally underwent tumor resection due to the shrinkage of tumor sizes after preoperative IM therapy. No postoperative complications occurred in our study group patients (Table 2).

**Table 2:** Main information of preoperative GIST patients

	General information			Preoperative IM therapy			Operation		
No.	G/A (years)	Tumor location	Mutation analysis	P/R	Dose (mg/day)	Duration (months)	Assessed before IM therapy	Performing after IM therapy	Margin status
1	F/50	Stomach	*KIT* E11 mut.	P	400	4	Total gastrectomy	Partial gastrectomy combined with spleen resection	R0
2	M/50	Stomach	*KIT* E11 mut.	P	400	6	Total gastrectomy	Partial gastrectomy (laparoscopic)	R0
3	M/48	Stomach	*KIT* E11 mut.	P	400	6	Total gastrectomy	Partial gastrectomy (laparoscopic)	R0
4	M/63	Stomach	*KIT* E11 mut.	P	400	3	Total gastrectomy	Partial gastrectomy	R0
5	F/65	Stomach	*KIT* E11 mut.	P	400	7	Total gastrectomy	Partial gastrectomy (laparoscopic)	R0
6	M/59	Stomach	Wild-type	P	400	4	Proximal gastrectomy	Proximal gastrectomy	R0
7	F/68	Stomach	*KIT* E11 and E13 mut.	P	400	7	Total gastrectomy	Partial gastrectomy	R0
8	M/58	Stomach and liver	*KIT* E11 mut.	R	400	8	Unresectable	Partial gastrectomy combined with spleen resection	R2
9	M/70	Stomach	*KIT* E11 mut.	P	400	12	Total gastrectomy	Partial gastrectomy	R0
10	M/58	Stomach	*KIT* E11 mut.	R	400	26	Unresectable	Partial gastrectomy	R0
11	F/44	Stomach	*KIT* E11 mut.	P	400	11	Total gastrectomy	Partial gastrectomy	R0
12	F/31	Stomach	*KIT* E11 mut.	R	400	8	Unresectable	Partial gastrectomy	R0
13	F/22	Stomach	*KIT* E11 mut.	P	400	10	Total gastrectomy	Partial gastrectomy	R0
14	F/40	Stomach	*KIT* E11 mut.	P	400	9	Total gastrectomy	Partial gastrectomy	R0
15	M/46	Small intestine	Wild-type	P	400	9	Pancreaticoduodenectomy	Pancreaticoduodenectomy	R0
16	F/53	Small intestine	*KIT* E9 mut.	P	600	4	Pancreaticoduodenectomy	Partial duodenum resection	R0
17	M/38	Small intestine	*KIT* E11 mut.	P	400	7	Pancreaticoduodenectomy	Pancreaticoduodenectomy	R0
18	M/67	Small intestine	*KIT* E9 mut.	R	600	10	Unresectable	Local resection of liver	R0
19	M/47	Small intestine	*KIT* E9 mut.	R	600	8	Unresectable	Tumor resection combined with partial small bowel resection	R0
20	M/65	Rectum	*KIT* E11 mut.	P	400	12	Abdominoperineal resection	Abdominoperineal resection (laparoscopic)	R0
21	M/48	Rectum	*KIT* E11 mut.	P	400	7	Abdominoperineal resection	Transanal minimally invasive surgery	R0
22	F/32	Rectum	*KIT* E11 mut.	P	400	9	Abdominoperineal resection	Sphincter-sparing resection	R0
23	M/54	Rectum	*KIT* E17 mut.	P	400	12	Abdominoperineal resection	Abdominoperineal resection (laparoscopic)	R0
24	M/46	Pelvic/retroperitoneal	*KIT* E11 mut.	R	400	18	Unresectable	Retroperitoneal tumor resection	R0
25	M/40	Pelvic/retroperitoneal	*KIT* E11 mut.	R	400	7	Unresectable	Pelvic tumor resection	R0

*GIST* gastrointestinal stromal tumor, *G/A* gender/age, *M* male, *F* female, *P/R* primary/recurrent and metastasis, *mut* mutation, *IM* imatinib mesylate

### Optimal duration of preoperative IM therapy

**Fig. 3:**
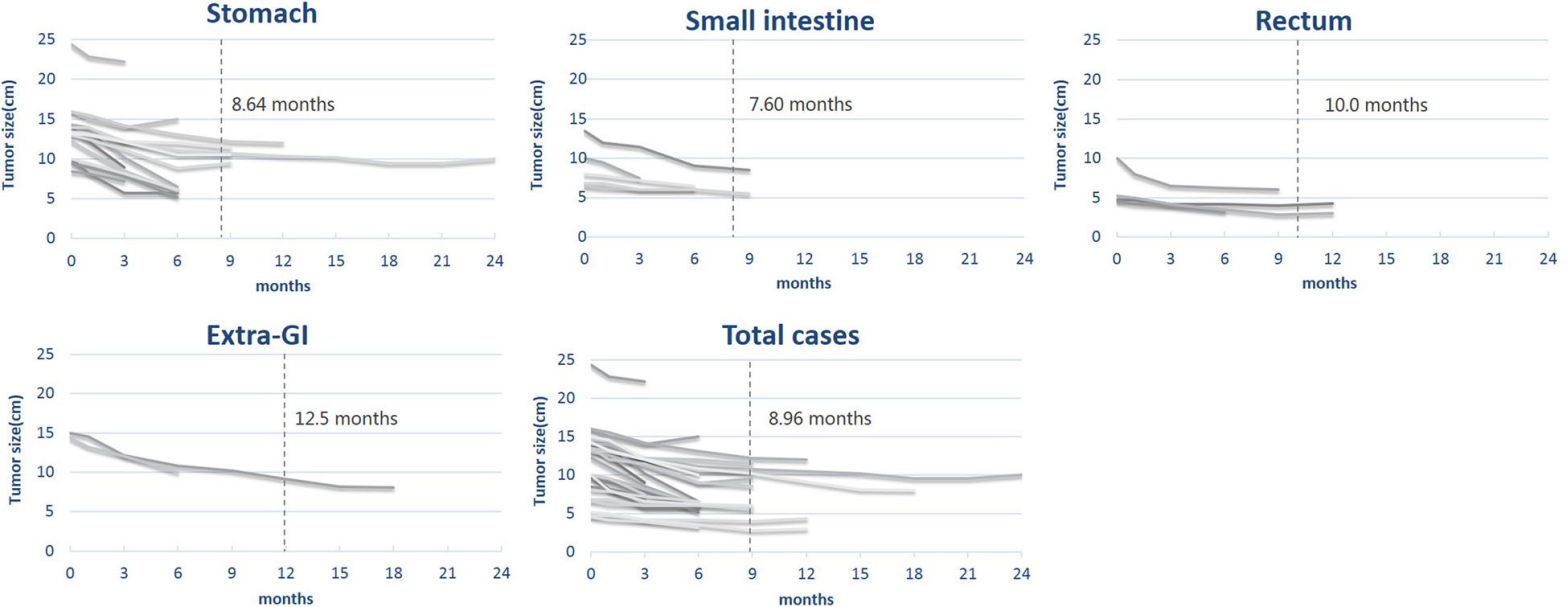
Change in tumor sizes after the initiation of IM therapy: stomach, small intestine, rectum, extra-GI, and total cases

We found that the duration of preoperative IM therapy was associated with tumor localization. Gastric GISTs was treated with a median duration of 8.64 months, and small intestine GIST patients underwent preoperative IM therapy with a median duration of 7.6 months; however, treatment for GISTs located in the rectum and pelvis/retroperitoneum seemed to have a longer duration, with a median duration of 10.0 months and 12.5 months, respectively. The total cases were administered preoperative IM therapy with a median duration of 8.96 months (Fig. 3).

We found that the mean duration of preoperative IM therapy was 8.96 months. To further investigate the data, we divided GIST patients into groups that received IM therapy for less than 8.96 months and more than 8.96 months. As shown in Table 3, we found that there were no significant differences in characteristics between the groups; however, it seems that tumors located in the stomach and small intestine had a shorter duration than those located in the rectum or pelvis/retroperitoneum, indicating that the upper gastrointestinal tract might have a better response and shorter duration of IM therapy. Due to the limited cases in our retrospective study, there were no significant differences between the two groups.

**Table 3:** Characteristics of GIST patients with different duration of preoperative IM therapy (*n* = 25)

Characteristics	Cases	Duration of IM therapy(months)		*P* value
		≤ 8.96	> 8.96	
Age (years) (mean ± SD)	25	51.29 ± 10.64	49.45 ± 15.06	0.725
Gender				0.973
Male	16	9	7	
Female	9	5	4	
Classify of preoperative therapy				1.000
Locally advanced GISTs	19	11	8	
Recurrent/metastasis GISTs	6	3	3	
Primary localization				0.350
Stomach + small intestine	19	12	7	
Rectum + pelvic/retroperitoneal	6	2	4	
Dose of IM therapy				1.000
400 mg/day	22	12	10	
600 mg/day	3	2	1	
Response to IM therapy (according toChoi criteria)				1.000
PR	24	13	11	
SD	1	1	0	

### Other influencing factors

In our study, including *KIT* exon 11, 13, and 17 mutation GISTs, which were truly well responsive to imatinib therapy at a dosage of 400 mg daily, *KIT* exon 9 mutation GISTs were also well responsive to imatinib therapy at a dosage of 600 mg daily, with obvious shrinkage of their tumor size. Two patients with wild-type GISTs showed variable responses to imatinib. One patient showed a good response at a dosage of 400 mg daily, and the other showed an SD response to imatinib. The latter patient underwent surgery after 4 months, received preoperative IM therapy, and finally showed recurrence at 26 months after surgery; sunitinib (50 mg daily) was administered immediately after recurrence was detected.

**Fig. 4:**
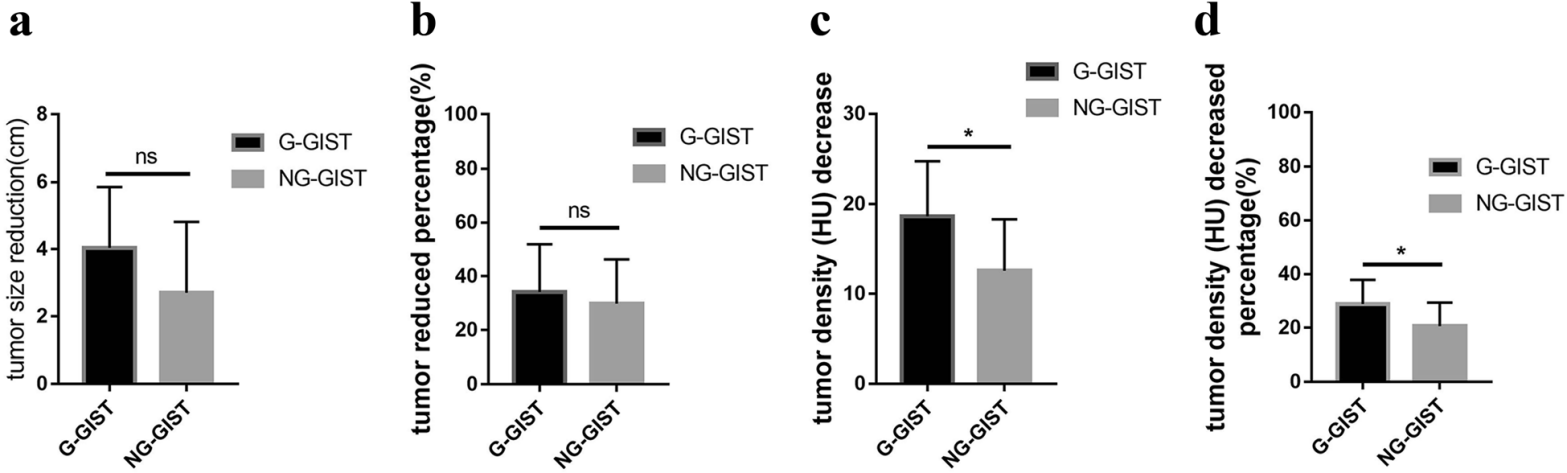
Response to preoperative IM therapy for gastric GISTs (G-GISTs) and non-gastric GISTs (NG-GISTs) (**a–d**): tumor size reduction, tumor-reduced percentage, tumor density (HU) decrease, and tumor density (HU) decreased percentage

Moreover, we investigated the influence of tumor location with IM therapy; we divided all GIST patients into gastric GIST (G-GIST) and non-gastric GIST (NG-GIST) groups. We found that the G-GIST group seemed to have a better tumor size reduction (4.04 ± 1.81 cm vs 2.70 ± 2.11, *P* = 0.102) and a reduced percentage (34.15% ± 17.77% vs 29.81% ± 16.41%, *P* = 0.537); however, we found that the G-GIST group achieved a better decrease in tumor density (HU) (18.64 ± 6.12 vs 12.54 ± 5.75, *P* = 0.018) and decreased percentage of tumor density (HU) (28.88% ± 8.94% vs 20.57% ± 8.82%, *P* = 0.030) (Fig. 4), which suggested that gastric GISTs had a better response to IM preoperative therapy than non-gastric GISTs.

### Prognosis of preoperative IM therapy patients

**Fig. 5:**
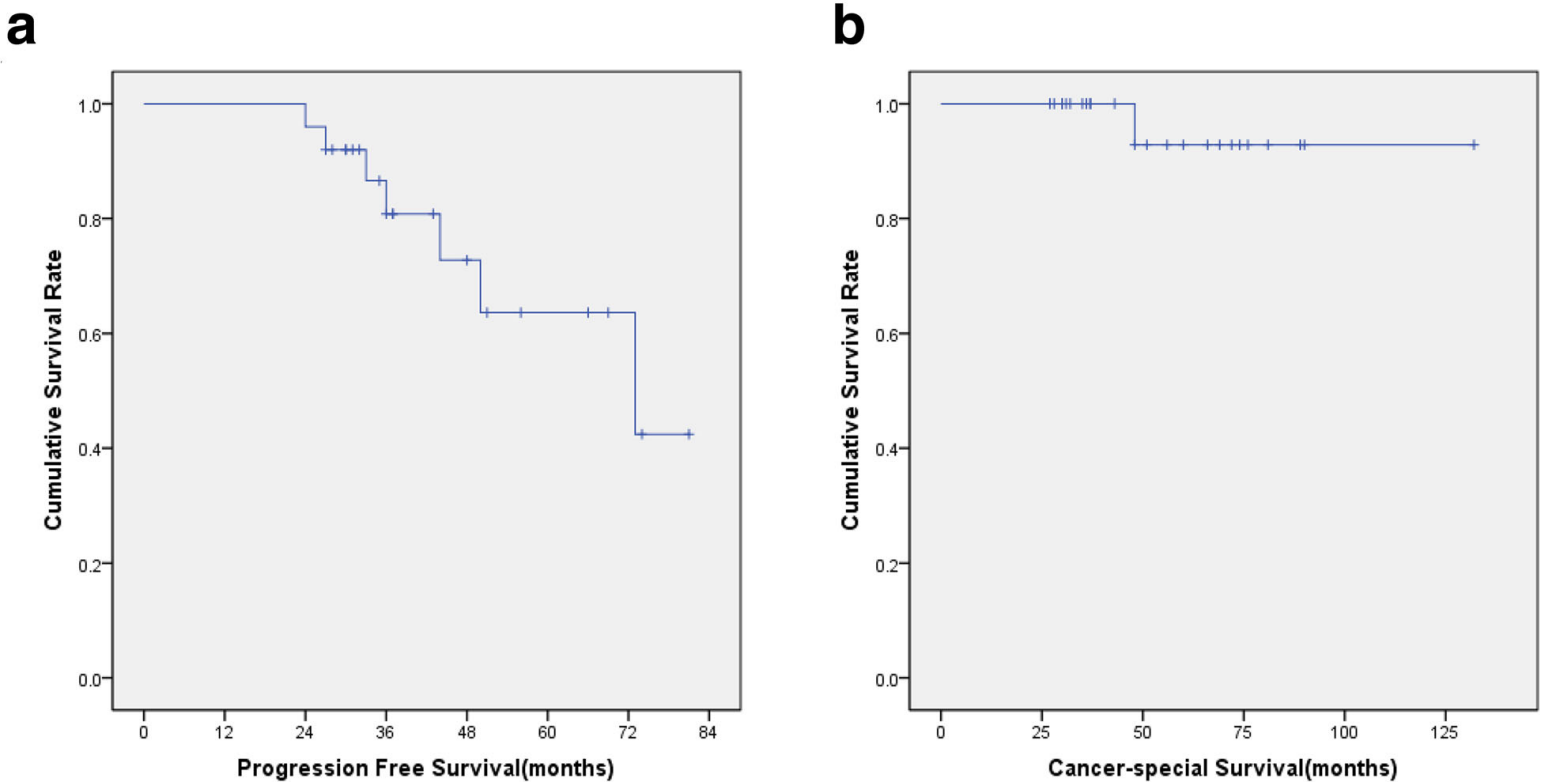
Progression-free survival (**a**) and cancer-specific survival (**b**) of patients treated with preoperative IM therapy, followed by surgery and postoperative IM therapy

The follow-up period ranged from 27 to 132 months with a median period of 55.12 months from the onset of preoperative IM therapy (Fig. 5). One patient failed to follow up at 48 months (42 months after surgery). The 2-year PFS and 5-year PFS were 92% and 60%, respectively, and the total 5-year cancer-specific survival (CSS) was 92%. One patient died at 48 months (41 months after surgery) because of tumor progression and refusal of any IM or other TKIs while exhibiting disease progression.

## Discussion

GIST is the most common mesenchymal tumor occurring in the digestive tract and originates from mesenchymal stem cells (interstitial cells of Cajal) [2]. With the unstable biological behavior of GISTs, and because traditional chemotherapy and radiotherapy seem to have no effect on GISTs, surgery may be the only way to cure this disease, in approximately 60% of GIST patients with localized GISTs [3]. In addition, up-front surgery for tumor resection should be deeply considered in cases requiring extensive organ resections for R0 resection, especially total gastrectomy, pancreaticoduodenectomy, or abdominoperineal excision, which might eventually lead to permanent changes in lifestyle or increase the incidence of surgical complications. Preoperative treatment is beneficial for those patients and for patients with unresectable GIST. In particular, mutation analysis has shown that TKIs are sensitive to IM treatment and can reduce the GIST cell population, which finally reduces the tumor size, may facilitate R0 resection, and can reserve the structure and function of target organs to the greatest extent [18]. Moreover, IM therapy before the operation can decrease the risk of bleeding, postoperative complications, and tumor rupture during the operation, leading to a lower possibility of tumor cell dissemination [19]. Several studies have shown that locally advanced, unresectable, and recurrent/metastatic GIST patients may benefit from preoperative adjuvant IM therapy. For example, Sumin Tang found that patients with preoperative IM treatment can receive more extensive R0 resection of tumors and lower surgical morbidity in large GISTs, with a median length of 12 months of preoperative IM treatment [20]. Tielen et al. found that preoperative IM treatment was feasible in locally advanced GISTs, with a median decrease of 50% in tumor size in 57 patients and a median IM duration of 32 weeks. Moreover, preoperative IM treatment combined with surgery improved PFS and OS in those patients [21]. Those studies recommended the feasibility and effectiveness of preoperative IM treatment; hence, the latest European Society for Medical Oncology (ESMO) and National Comprehensive Cancer Network (NCCN) guidelines recommended preoperative IM treatment as an effective treatment for these special patients [22, 23]. However, there is still controversy regarding which Lee et al. indicated that caution should be taken when dealing with preoperative IM therapy because it might jeopardize anastomotic healing of the bowel and increase the postoperative complications in those patients [24]. In our study, we found that 25 enrolled patients, including 18 locally advanced GISTs and 7 recurrent/metastatic GISTs, responded well to preoperative IM treatment and had 1 SD status and 24 PR status, with 96% patients having a decrease in tumor size. Moreover, all the patients well tolerated IM therapy, and none of them had complications after tumor resection, indicating that preoperative IM therapy was still feasible for locally advanced GISTs and recurrent/metastatic GISTs, especially for patients with unresectable tumors or needing extensive R0 organ resection.

Gene mutation analysis plays a critical role in the patient plan for IM therapy because patients with advanced and recurrent/metastasis have different responses to imatinib, and the aim of preoperative IM therapy is to downstage the current situation of GIST patients, which depends on the responses to IM therapy. Gene mutation analysis can help clinicians formulate individualized precision-medicine plans for GIST patients, and most GISTs have *KIT* mutations occurring in exons 9, 11, 13, and 17 and *PDGFRA* mutations occurring in exons 12, 14, and 18 [8, 9]. Patients with *KIT* exon 11 mutations, including deletion and duplication, seem to benefit most and respond well to imatinib, so it is generally advised that at a dosage of 400 mg daily, European patients with *KIT* exon 9 mutations are still sensitive to imatinib but need 400 mg twice daily [8, 22, 23]. However, Chinese patients are usually intolerant, and as a consequence, the dosage of IM in China is 600 mg administered daily [25], and *KIT* exon 13 and 17 mutations are rare in GISTs. Limited case reports have shown that *KIT* exon 13 and 17 mutations seem likely to respond to imatinib therapy. Engin et al. analyzed a familial GIST with 3 *KIT* exon 13 mutation patients and found that they responded well to imatinib adjuvant therapy at a dosage of 400 mg daily [26]. Similar findings have been found by Heinrich et al. [27] and Debiec-Rychter et al. [28]. Patients with *KIT* exon 17 mutations can rarely be detected in GISTs, and their response to imatinib is variable; in most cases, they respond well to imatinib therapy [29–31]. In addition, approximately 10% of GIST patients have no mutation in either *KIT* or *PDGFRA*, and these tumors are called wild-type GISTs and have an approximately 0% to 45% likelihood of a response to imatinib [8, 23]. To date, all the reported phase II trials of preoperative treatment did not include mutational analysis as a prerequisite for neoadjuvant treatment. In our study, we tested all the gene mutations in GIST patients who were going to take preoperative IM therapy, and the results showed that GISTs with *KIT* exon 11, 13, and 17 mutations truly responded well to imatinib therapy at a dosage of 400 mg daily. GISTs with *KIT* exon 9 mutations also responded well to imatinib therapy at a dosage of 600 mg daily, with obvious shrinkage of their tumor size. Two patients with wild-type GISTs showed variable responses to imatinib. One patient showed a good response at a dosage of 400 mg daily; the other patient showed an SD response to imatinib, underwent surgery after 4 months of preoperative IM therapy, and finally exhibited recurrence at 26 months after surgery and received sunitinib (50 mg daily) immediately after the recurrence was detected. Our results indicated that mutation analysis is strongly recommended before preoperative IM therapy in order to formulate an individualized and precision medicine plan for the patients.

The optimal duration of preoperative IM therapy remains controversial because the optimal operation window might be missed if patients take long-term imatinib therapy, which would finally lead to resistance to IM therapy and promote tumor progression. Short-term imatinib therapy generally achieves a finite efficient shrinkage of tumor size. To date, several studies have been performed to probe the appropriate duration of preoperative IM therapy; these results are inconsistent, and the duration varies considerably. The RTOG 0132/ACRIN 6665 clinical trial including 31 primary GIST patients and 22 recurrent/metastatic GIST patients performed effective preoperative IM treatment after a duration of 8 to 12 weeks (median, 9.9 weeks) at a dosage of 600 mg daily, and the 5-year PFS and OS were 46.1% and 73.6%, respectively [32]. Another phase III study (B2222) indicated that most preoperative IM therapies for unresectable/metastatic GISTs required a duration of 5.3 months to obtain a good response [33]. A Japanese phase II study (STI571B1202) proved that a plateau response of preoperative IM therapy for unresectable/metastatic GISTs needs 200 days [34]. With such studies focusing on the optimal duration of preoperative IM therapy, the latest ESMO guidelines recommend a duration of 6 to 12 months while initiating IM therapy and following surgical intervention [22]. The NCCN guidelines recommend a duration of at least 6 months to obtain the maximal response [23]. In our study, we performed surgery after a median time of 8.96 months to obtain a plateau response (ranging from 3 to 26 months), which was longer than that in the RTOG 0132/ACRIN 6665 clinical trial and B2222 trial but similar to that in the STI571B1202 study. We also found that different locations had their respective durations and plateau responses to IM therapy, as follows: extra-GI (12.5 months), rectum (10.0 months), stomach (8.64 months), and small intestine (7.60 months). In addition, even one patient remained sensitive to IM after 26 months of initiation preoperative IM therapy. Hence, there are individual differences at the duration of preoperative IM therapy, but most achieved the best beneficial response at a median duration of 8.96 months.

Interestingly, we found that tumor location in the upper gastrointestinal tract, especially in the stomach, had a better response to preoperative IM therapy than did non-gastric GISTs. A Japanese-Korean UMIN00000003114 study included gastric GISTs larger than 10 cm, and other localizations were not included, with 400 mg imatinib for 6–9 months. The results showed that none of the 53 patients developed tumor progression, 62% achieved a PR response, and all patients achieved a good response to preoperative IM therapy, suggesting that neoadjuvant imatinib therapy can be beneficial for larger gastric GISTs, which was similar to our research [35]. However, few studies have focused on the influence of tumor localization in preoperative IM therapy. Our results recommended that gastric GISTs might have a better response to preoperative IM therapy, and if tumors were non-gastric, especially in the rectum, the response might be worse, and a longer duration was suggested for patients undergoing preoperative IM therapy.

It should be stressed that, first, this was a retrospective study, so we cannot compare the outcomes of patients undergoing extensive surgery without preoperative IM therapy to those of patients undergoing surgery with neoadjuvant IM therapy, as ESMO and NCCN guidelines recommend that neoadjuvant IM therapy provides a benefit and a better quality of life for those patients. Second, our study group was a small-scale sample, and our study was a non-randomized controlled trial, so it is difficult to obtain any definitive conclusion, including the exact, optimal duration of preoperative IM therapy. Further randomized controlled trials and prospective studies should be performed. From our recent study results, we suggest that preoperative IM therapy is a recommendable strategy for locally advanced and recurrent/metastatic GISTs.

## Conclusion

Preoperative IM therapy is a feasible method for locally advanced and recurrent/metastatic GISTs. Mutation analysis should be performed before IM therapy, and the initial dosage of IM should be based on the results of the mutation analysis. Although there are individual differences in the duration of preoperative IM therapy, it mostly has a median time of 8.96 months. Moreover, gastric GISTs have a better response to preoperative IM therapy than do non-gastric GISTs.

## Supplementary information

**Additional file 1: Table S1.** Detail information of recurrent/metastatic GIST patients.

## Data Availability

The raw data underlying this paper are available upon request to the corresponding author due to ethical restrictions.
