# Supplementary material for: Preoperative adjuvant therapy for locally advanced and recurrent/metastatic gastrointestinal stromal tumors: a retrospective study
Source: World J Surg Oncol. 2020 Apr 7;18:70. doi: 10.1186/s12957-020-01840-9 (PMC7140320; doi:10.1186/s12957-020-01840-9)
Supplement: Supplementary file 1 — Additional file 1: Table S1. Detail information of recurrent/metastatic GIST patients. [file 12957_2020_1840_MOESM1_ESM.docx]

| Table S1. Detail information of recurrent/metastatic GIST patients. | | | | | |
| --- | --- | --- | --- | --- | --- |
|  | General information | | Detail information | | |
| No. | G/A  (years) | Tumor location | R/M | Metastatic location | Margin  status |
| 8 | M/58 | Stomach and liver | M | Liver | R2 |
| 10 | M/58 | Stomach | R | - | R0 |
| 12 | F/31 | Stomach | R | - | R0 |
| 18 | M/67 | small intestine | M | Liver | R0 |
| 19 | M/47 | small intestine | R | - | R0 |
| 24 | M/46 | Pelvic/retroperitoneal | M | Retroperitoneum | R0 |
| 25 | M/40 | Pelvic/retroperitoneal | M | Pelvic cavity | R0 |
| GIST: gastrointestinal stromal tumor, G/A: Gender/age, M: male, F: female, R/M: recurrent/metastasis, mut: mutation, IM: Imatinib Mesylate. | | | | | |
